# Detection of Methicillin-Resistant Staphylococci Isolated from Food Producing Animals: A Public Health Implication

**DOI:** 10.3390/vetsci3030014

**Published:** 2016-07-04

**Authors:** Etinosa O. Igbinosa, Abeni Beshiru, Lucy U. Akporehe, Abraham G. Ogofure

**Affiliations:** Applied Microbial Processes & Environmental Health Research Group, Department of Microbiology, Faculty of Life Sciences, University of Benin, Private Mail Bag 1154, Benin City 300001, Nigeria; AB-bash_ab@rocketmail.com (A.B.); LUA-agimlucy7@gmail.com (L.U.A.); AGO-abraham.ogofure@uniben.edu (A.G.O.)

**Keywords:** multi-drug resistant, staphylococcal, resistance gene, virulence gene, infectious disease

## Abstract

The emergence of antibiotic-resistant bacteria in food animals is a potential public health concern. Staphylococci are a significant opportunistic pathogen both in humans and dairy cattle. In the present study, the genotypic characterization of methicillin-resistant staphylococcal strains recovered from dairy cattle in a rural community (Okada, Edo State, Nigeria) was investigated. A total of 283 samples from cattle (137 milk samples and 146 nasal swabs) were assessed between February and April 2015. Antimicrobial susceptibility was performed by Kirby-Bauer disc diffusion method. Polymerase chain reaction (PCR) assay was employed for the detection of 16S rRNA, *mec*A and Panton-Valentine Leucocidinis (PVL) genes. The staphylococcal strains were identified through partial 16S ribosomal ribonucleic acids (rRNA) nucleotide sequencing, and Basic Local Alignment Search Tool (BLAST) analysis of the gene sequence showed that the staphylococcal strains have 96%–100% similarity to *Staphylococcus aureus* (30), *S. epidermidis* (17), *S. haemolyticus* (15), *S. saprophyticus* (13), *S. chromogenes* (8), *S. simulans* (7), *S. pseudintermedius* (6) and *S. xylosus* (4). Resistance of 100% was observed in all *Staphylococcus* spp. against MET, PEN, CLN, CHL and SXT. Multi-drug resistant (MDR) bacteria from nasal cavities and raw milk reveals 13 isolates were MDR against MET^R^, PEN^R^, AMX^R^, CLN^R^, CHL^R^, SXT^R^ CLX^R^, KAN^R^, ERY^R^, and VAN^R^. Of all isolates, 100% harboured the *mec*A gene, while 30% of the isolates possess the PVL gene. All *S. aureus* harboured the PVL gene while other *Staphylococcus* spp. were negative for the PVL gene. The presence of methicillin-resistant *Staphylococcus* spp. isolates in dairy cattle is a potential public health risk and thus findings in this study can be used as a baseline for further surveillance.

## 1. Introduction

Staphylococci are frequently found amongregular bacterial flora of the flexible continuous coverings of human andother animal bodies, and mucosal surfaces of the respiratory, upper alimentary, and urogenital tracts of mammals and birds. In milk-producing animals, *Staphylococcus* species still remains one of the most indicative organisms connected with clinical and sub-clinical bovine mastitis worldwide [1]. *Staphylococcus* spp. have been the principal theme of studies on antibiotic resistance because of their significance for all forms of milk-producing animals [2]. Nevertheless, in recent years, the rate of reported coagulase negative staphylococci (CNS) mastitis has become substantially more numerous [2]. In human medicine, antimicrobial multi-resistance is rapidly encountered and methicillin-resistant *S. aureus* (MRSA) and methicillin-resistant coagulase-negative *Staphylococcus* (MR-CNS) strains are among the most threatening bacteria involved in nosocomial infections. In veterinary science, staphylococcal strains play a role in a wide range of diseases in various animal species, such as bovine mastitis (e.g., *S. aureus* and coagulase-negative staphylococcal strains such as *S. epidermidis*, *S. haemolyticus*, *S. chromogenes*), canine pyoderma (*S. pseudintermedius*), exudative epidermitis in pigs (*S. hyicus*), or septicaemia in poultry (*S. aureus)* [3]. 

Strains of MRSA are resistant to β-lactam antibiotics, including all penincillinase-stable β-lactam antibiotics. Resistance is most commonly depicted by *mec*A gene, which is localized on a genomic island known as the staphylococcal cassette chromosome (SCCmec) [4]. This gene encodes for penicillin-binding protein (PBP2a) which localizes in the bacterial cell wall and has a low affinity for β-lactam antibiotics. Therefore, this group of antibiotics is not effective against bacteria expressing *mec*A gene. Due to the disseminated use of antibiotics, the resistance profile of microorganisms is increasing within bacterial population. Community-acquired MRSA is now increasingly identified with no prior hospital exposure. These community-based infections have been reported in patients from both rural and urban settings [1].

Staphylococci entails diversity of disease in humans and animals, its ability to cause diseases is predominantly connected to a combination of toxin-mediated virulence, invasive capacity, and antibiotic resistance [5]. In 2009, the European Food Safety Authority (EFSA) underlined the increasing worry for public health represented by the presence of methicillin-resistant *S. aureus* (MRSA) in food-producing animals and suggested that further work should be carried out on sampling detection and quantification of MRSA carriage in both humans and animals, as well as on the infection of food and the environment [6]. In the present study we investigated the occurrence and characterization of methicillin-resistant staphylococcal isolates from free-range dairy cattle in a rural community Okada, Edo State, Nigeria.

## 2. Materials and Methods

### 2.1. Study Design

A cross-sectional study design was used to determine the bacteriological analysis of raw milk and nasal cavities in a rural community Okada, Edo State of Nigeria. 

### 2.2. Sampling and Isolation Method

A total of 283 samples were examined and assessedfrom free-range dairy cattle (137 milk samples and 146 nasal swabs) between February and April 2015 in a rural community in Okada, Edo State, Nigeria. This research was carried out in a predominantly Edo South region (Okada, Ovia North-East Local Government Area, Edo State, Nigeria) population where electricity and formal schooling are rare in the pastoral and rural communities. People in the region live in close proximity to livestock and the livestock herds in the area frequently come in contact with one another. In the communities being studied, livestock antibiotics are mostly administered by animal owners rather than veterinarians.

Sampling was performed using standard procedures by the European Food Safety Authority [6]. Prior to sample collection, the udder, teats, and adjacent flank areas were thoroughly washed and dried with single-service sanitary paper towel and the teats were disinfected with 70% alcohol. Fifteen millilitres (15 mL) of milk from each quarter was collected. The milk samples were transported in an ice box to the laboratory within 3 h. Ethics approval was not required for this study as taking a nasal swab does not cause pain, distress or lasting harm. MRSA was isolated using 100 mL Mueller Hinton (MH) broth supplemented with NaCl (6.5%) and incubated at 37 °C for 20–24 h. One millilitre (1 mL) of Mueller Hinton (MH) broth was added to Tryptic Soy Broth (TSB) and incubated overnight at 37 °C [7]. Ten micro-litres (10 μL) of Tryptic Soy Broth (TSB) were spread on MRSA selective agar plate (CHROMagar^TM^ MRSA-ITK Diagnostics BV, Netherlands) and incubated for 48 h at 37 °C. At both 24 and 48 h, plates were inspected and suspected colonies were purified on Columbia agar plates with 5% sheep blood (CSB) and incubated overnight at 37 °C. All isolates were confirmed to be *Staphylococcal aureus,* characteristically: by their typical colonial appearance (displaying golden-yellow colonies), having a positive catalase test, and staining as Gram-positive cocci in clusters. Other staphylococci species were culturally and morphologically identified as coagulase-negative staphylococci (CNS) forming non-golden yellow and non-beta-hemolytic colonies.

### 2.3. Bacterial Identification of Staphylococci

Staphylococcal colonies were identified by standard microbiological culture-based tests which included Gram-staining, catalase testing (using 3% hydrogen peroxide), indole, oxidase, coagulase, citrate, urease, Voges-prosakaser, slide agglutination test (BBL™ Staphyloslide™), DNase test, sugar fermentation, and the oxidation and fermentation of mannitol salt agar [8]. All tests were performed according to standard guidelines and *S. aureus* (ATCC 29213) was used as a positive control in each test protocol.

### 2.4. Molecular Identification by PCR

#### 2.4.1. Genomic DNA Extraction

Isolation of genomic DNA extraction was carried out as described by Shuiepet et al. [9]. Single colonies grown on Brain Heart Infusion agar were transferred to 1.5 mL of Brain Heart Infusion broth and cultures were grown on a shaker for 48 h at 28 °C. After this period, cultures were centrifuged at 4600 rpm for 5 min. The resulting pellets were re-suspended in 520 µL of TE buffer, then 15 µL of 20% SDS and 3 µL of Proteinase K (20 mg·mL^–1^) were added. The mixture was incubated for 1 h at 37 °C, then 100 µL of 5 M NaCl and 80 µL of a 10% cetyltrimethyl ammonium bromide (CTAB) solution in 0.7 M NaCl were added and mixed. The suspension was incubated for 10 min at 65 °C and kept on ice for 15 min. An equal volume of chloroform: isoamyl alcohol (24:1) was added, followed by incubation on ice for 5 min and centrifugation at 7200 rpm for 20 min. The aqueous phase was transferred to a new tube, isopropanol (1:0.6) was added and DNA was precipitated at −20 °C for 16 h. DNA was collected by centrifugation at 7200 rpm for 10 min, washed with 500 μL of 70% ethanol, air-dried at room temperature for approximately 3 h and finally dissolved in 50 μL of TE buffer. The genomic DNA was stored at −20 °C and used for molecular identification and bacterial typing protocols.

#### 2.4.2. PCR for Identification of Staphylococcal Isolates

PCR identification of *Staphylococcus* was carried out as described by Ma et al. [10]. The PCR consisted of a final volume of 50 µL which included 8 µL DNA and 42 µL reaction consisting of 5×GoTaq green reaction, 10 mM of each dNTP, 10 pmol each 27F: 5’-AGAGTTTGATCMT GGCTCAG-3’ and 1525R: 5’-AAGGAGGTGWTCCARCC-3’ specific for ~1500 bp conserved domain of the 16S rRNA gene of bacteria and 0.3 units of Taq DNA polymerase. PCR was carried out using the following thermal cyclic regime: an initial denaturation at 94 °C for 1 min, followed by 29 cycles of denaturation at 94 °C for 30 s, annealing at 50 °C for 1 min, an extension at 72 °C for 1.5 min, a final extension of 72 °C for 5 min, and cooling to 4 °C. Electrophoresis of amplicons was performed with 1% agarose gel containing ethidium bromide (EtBr) 0.5 mg·L^−1^, for 1 h at 100 V in 0.5 × TAE buffer (40 mM Tris-HCl, 20 mM Na-acetate, 1 mM EDTA, pH 8.5) and visualized under a UV transilluminator.

#### 2.4.3. Sequencing of the 16S rRNA Genes

Sequencing was carried out as described by Tamura et al. [11]. The purified DNA samples were sequenced with an Automated DNA sequencing Analyzer (ABI 3730X) using 27F and 1525R primers. All DNA sequences were subjected to the method of Altschul et al. [12] for comparison with the Basic Local Alignment Search Tool (BLAST) program alignment tool from GenBank at the National Center for Biotechnology Information.

#### 2.4.4. Specific PCR for the Identification of Methicillin-Resistant *Staphylococcus aureus*

The detection of the *mec*A gene was carried out as described by Ahmed et al. [13]. The 25.0 μL volume of PCR reaction mixture contained 1.0 μL of genomic DNA, 12.5 μL of PCR Master mix 7.5 μL PCR H_2_O and 2μL each of *mec*A primers. *mec*A gene was amplified with the following primers: *mec*A-F: (5’-TCCAGATTACAACTTCACCAGG-3’); *mec*A-R: (5’-CCACTTCATATCTTGTAACG-3’) with amplicon size 162bp. DNA amplification was carried out for 40 cycles according to the following protocol: denaturation at 94 °C for 30 s, annealing at 55 °C for 30 s, extension at 72 °C for 1 min with a final extension at 72 °C for 5 min, and cooling to 4 °C. Electrophoresis of amplicons was performed with 1% agarose gel containing ethidium bromide (EtBr) 0.5 mg·L^−1^, for 1 h at 100 V in 0.5 × TAE buffer (40 mM Tris-HCl, 20 mM Na-acetate, 1 mM EDTA, pH 8.5) and visualized under a UV transilluminator. 

#### 2.4.5. Detection Luk S/F gene Sequences that Encode Virulence Determinants in Staphylococci

This was conducted according to the protocol described by McDonald et al. [14]. The 25.0 μL volume of PCR reaction mixture contained 1.0 μL of genomic DNA, 2.0 μL each of lukS-PV and lukF-PVL primers, 12.5 μL of Red Taq Master mix and 7.5 μL PCR H_2_O. Primers were luk-PV-1(5’-ACACACTATGGCAATAGTTATTT-3’) and luk-PV-2(5’-AAAGCAATGCAATTGAT GTA-3’) with amplicon size 176bp. Amplification was carried out for 40 cycles according to the following protocol: denaturation at 94 °C for 30 s, annealing at 55 °C for 30 s, extension at 72 °C for 1 min with a final extension at 72 °C for 5 min, and cooling to 4 °C. Electrophoresis of amplicons was performed with 1% agarose gel containing ethidium bromide (EtBr) 0.5 mg·L^−1^, for 1 h at 100 V in 0.5 × TAE buffer (40 mM Tris-HCl, 20 mM Na-acetate, 1 mM EDTA, pH 8.5) and visualized under a UV transilluminator.

### 2.5. Determination of the Antibiotic Resistance Profiles of Staphylococcal Isolates

#### 2.5.1. Antibiotic Susceptibility Testing

The staphylococcal isolates that were positively identified using the culture-based methods were subjected to antibiogram characterization. All the bacterial isolates were tested for resistance or sensitivity to different antibiotics using the standard disc diffusion method (Kirby Bauer test) [15]. For the disc diffusion assay, bacteria were grown between 18 and 24 h on Mueller-Hinton agar, harvested and then suspended in 0.85% sterile physiological saline solution adjusted to a 0.5 McFarland turbidity standard, corresponding to 10^8^ cfu·mL^−1^. The inoculum was streaked onto plates of Mueller-Hinton agar using a sterile cotton swab and impregnated with appropriate antibiotics (Supplemental Material Table S1). The results were recorded after 24 h of incubation at 37 °C. Commercially available antibiotics discs, obtained from Mast Diagnostics, Merseyside, United Kingdom, were used to determine the resistance patterns of the isolates against 11 different antibiotics (1 dose/disc), grouped into 7 different classes of antibiotics. The diameter of the zone of inhibition around each disc was measured and interpreted as Resistant (R), Intermediate resistant (I) or Sensitive (S) in accordance with the recommended standard established by the Clinical Laboratory Standards Institute [16].

#### 2.5.2. Statistical Analysis

All data were analyzed using SPSS statistics, version 16.0 (IBM^®^ Corporation, Armonk, NY, USA). Antibiotic resistance and virulence factors of nasal cavity and raw milk isolates were compared using the analysis of variance (ANOVA), student’s T-test and the Regression test. The *p*-values of <0.05 were considered statistically significant.

## 3. Results

### 3.1. Specific PCR for the Identification of Staphylococcal Isolates

Among the total of 78 and 75 isolated presumptive staphylococcal isolates from nasal and raw milk samples, respectively-identified by cultural, morphological and biochemical characteristics-50 methicillin-resistant staphylococcal isolates each from the nasal cavity and the raw milk (100) were further confirmed using the 16S rRNA primer employing PCR assay which was more sensitive for confirmation of the isolates. The PCR products of the 16S rRNA for the confirmation of the 100 isolates from the nasal and raw milk samples and the partial sequence of the *Staphylococcus* species are shown in Supplemental Material Tables S2 and S3.

### 3.2. Prevalence and Species Diversity of Staphylococcal Isolates

Species diversity of staphylococcal isolates recovered from nasal and milk samples revealed 50 methicillin-resistant staphylococcal positive isolates from the nasal cavity and 50 methicillin-resistant staphylococcal strains from the raw milk samples (Table 1). The occurrence from the 100 positive methicillin-resistant staphylococcal isolates from the samples were: *S. aureus* (30: 19 nasal cavity and 11 raw milk), followed by *S. epidermidis* (17: 13 nasal cavity and 4 raw milk), *S. haemolyticus* (15:0 nasal cavity and 15 raw milk), *S. saprophyticus* (13:8 nasal cavity and 5 raw milk), *S. chromogenes* (8: 0 nasal cavity and 8 raw milk), *S. simulans* (7:0 nasal cavity and 7 raw milk), *S. pseudintermedius* (6:6nasal cavity and 0 raw milk) and *S. xylosus* (4:4 nasal cavity and 0 raw milk) (Table 1). Statistical analysis revealed that there was no significant difference observed in the distribution of staphylococcal isolates obtained from nasal and raw milk samples (*p* > 0.05).

### 3.3. Antibiotic Resistance of Staphylococcal Isolates from Nasal and Raw Milk Samples

Antimicrobial susceptibility profile of the *Staphylococcus* spp. showed that all the *Staphylococcus* spp. were 100% resistant to methicillin, penicillin, amoxicillin, clindamycin, chloramphenicol, and trimethoprim-sulfamethaxazole. *S. aureus*, *S. chromogenes*, *S. epidermidis*, *S. haemolyticus*, *S. simulans,* and *S. pseudintermedius* were 100% resistant to cloxacillin while *S. saprophyticus* and *S. xylosus* were 92.30% and 75% resistant to cloxacillin, respectively. *S. haemolyticus*, *S. simulans*, *S. saprophyticus,* and *S. xylosus* were 100% resistant to erythromycin while *S. aureus*, *S. chromogenes*, *S. epidermidis,* and *S. pseudintermedius* were 90%, 87.50%, 88.20% and 83.33% resistant to erythromycin, respectively. More so, *S. chromogenes*, *S. haemolyticus*, *S. simulans*, *S. pseudintermedius,* and *S. xylosus* were 100% resistant to kanamycin while *S. aureus*, *S. epidermidis,* and *S. saprophyticus* were 90%, 94.12% and 92.31% resistant to kanamycin, respectively (Table 2). Statistical analysis revealed that there was no significant difference observed between the antimicrobial susceptibility profiles of staphylococcal isolates (*p* > 0.05).

### 3.4. Multiple Antibiotic Resistance Phenotypes of Staphylococcal Isolates

Multi-drug resistant (MDR) isolates from nasal cavities and raw milk revealed that 100 of the isolates were resistant to MET^R^, PEN^R^, CLN^R^, CHL^R^, and SXT^R^; 97 isolates were resistant to MET^R^, PEN^R^, AMX^R^, CLN^R^, CHL^R^, SXT^R^, and CLX^R^; 91 isolates were resistant to MET^R^, PEN^R^, AMX^R^, CLN^R^, CHL^R^, SXT^R^, CLX^R^, and KAN^R^; 87 isolates were resistant to MET^R^, PEN^R^, AMX^R^, CLN^R^, CHL^R^, SXT^R^, CLX^R^, KAN^R^,and ERY^R^; 13 isolates were resistant to MET^R^, PEN^R^, AMX^R^, CLN^R^, CHL^R^, SXT^R^, CLX^R^, KAN^R^, ERY^R^,and VAN^R^ (Table S3). Statistical analysis revealed that there was a significant regression (*r* = 0.739) of the isolates on the antibiotics used (*p* < 0.01). This could be attributed to the incorporation of antibiotics in the feed of the cattle coupled with the administration of antimicrobial agents orally for prophylactic and therapeutic purposes. The appearance of resistance to multiple antimicrobial agents in staphylococcal strains has become a significant public health threat as there are few effective antimicrobial agents available for infections caused by these bacteria. As such, a joint initiative by the European Centre for Disease Prevention and Control (ECDC) and the Centers for Disease Control and Prevention (CDC) to create a standardised international terminology with which to describe acquired resistance profiles in bacterial isolates that are prone to MDR became a necessity. Non-susceptibility to ≥1 agent in ≥3 antimicrobial categories was considered MDR [17].

### 3.5. Detection of Resistance and Virulence Genes in Staphylococcal Isolates

**Figure 1:**
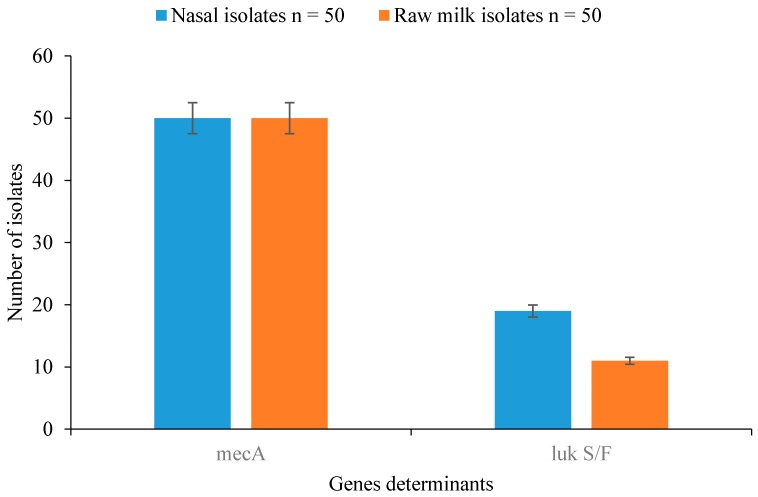
Detection of resistance and virulence genes in *Staphylococcus* spp. isolated from nasal cavity and raw milk samples.

**Figure 2:**
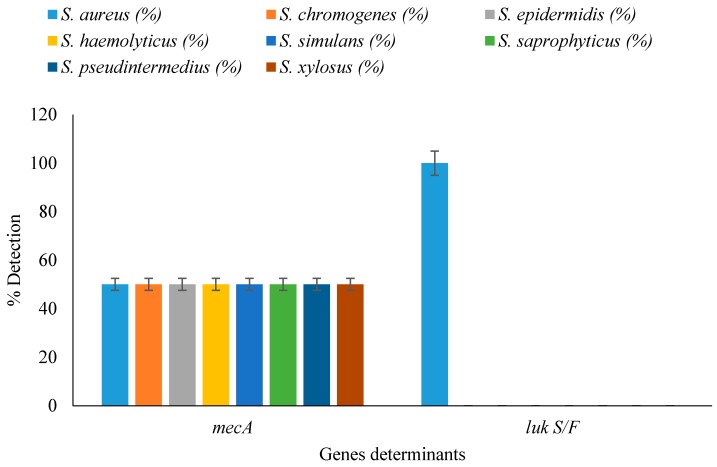
Detection of resistance and virulence genes in staphylococcal isolates from nasal and milk samples.

Detection of resistance- and virulence-determinant genes from nasal cavity and raw milk samples revealed that all isolates harboured the *mec*A gene, while 19/50 (38%) and 11/50 (22%) of the isolates from nasal and milk samples harboured thePanton-Valentine Leucocidinis (PVL) gene and possessed the virulence determinant (Figure 1). Of the *Staphylococcus* spp., all *S. aureus* amplified the PVL gene while other *Staphylococcus* spp. were negative for the PVL gene (Figure 2).

## 4. Discussion

*Staphylococcus* species have been reported in food animals as well as inwild and domestic birds. Some animals are asymptomatic while others suffer respiratory, gastrointestinal, or skin and soft tissue infections. *Staphylococcus aureus* is a significant cause of mastitis in cows and small ruminants [18]. A noted property of staphylococcal strains is their ability to become resistant to antimicrobials. The most clinically relevant staphylococcal strains in veterinary medicine are *Staphylococcus aureus* and members of the coagulase-negative staphylococcal group, particularly *Staphylococcus pseudintermedius*, *Staphylococcus epidermidis*, *Staphylococcus haemolyticus,* and *Staphylococcus saprophyticus* [19].

Resistance to methicillin and vancomycin in staphylococcal strains obtained from cow milk samples was reported by Pehlivanoglu and Yardimci [20]. Resistance to vancomycin among staphylococcal strains is very rare and, as such, it needs to be investigated further by assessing the minimum inhibitory concentration (MIC) values by broth microdilution test, as recommended by the CLSI and the EUCAST guidelines. Nasal carriage rates of methicillin-resistant *S. aureus* (MRSA) in cattle, and the resistance rates of methicillin-resistant *S. aureus* isolates against various antibiotics were reported by Garipcin and Seker [21]. The findings of Abd-Al-Azeem et al. [22] revealed that the highest resistance of the isolated strains of β-lactam resistant *S. aureus* was recorded for penicillin and the lowest was recorded for amoxicillin/clavulanate. This agrees with the antimicrobial susceptibility pattern of isolates in our study as all isolates were resistant to penicillin. According to the antibiotic susceptibility test findings by Garipcin and Seker [21], resistance to clindamycin, erythromycin, fusidic acid, mupirocin, rifampicin, and teicoplanin was determined in all three methicillin-resistant *S. aureus* strains isolated from cattle nasal samples. This agrees with the resistance to clindamycin and erythromycin as used in ourstudy (Table 2). In another study by Jahan et al. [23], the isolates from raw milk werefound to be resistant to penicillin (100%), erythromycin (75%), and amoxicillin (100%). On the other hand, the isolates were sensitive to ciprofloxacin (83.33%), oxacillin (100%), cloxacillin (100%), and neomycin (100%). This concursto large extent in our study, as the isolates from raw milk werefound to be resistant to penicillin (100%), erythromycin (98%), amoxicillin (100%), and cloxacillin (98%) (Table 2).

Methicillin resistance in staphylococcal strains can be transmitted between animals, to humans in contact with them, and also to the environment. It has been documented that the glycopeptide-resistant enterococci have emerged after using an antibiotic-avoparcin-as growth promoter in food animals [24]. Resistance to glycopeptides is a cause for concern, as vancomycin-a glycopeptides-is the drug of choice for the treatment of infections as a result of multi-drug resistant staphylococcal strains. Similarly, the use of tylosin in animal feed supplement has resulted in the development of erythromycin-resistant staphylococcal strains. Use of enrofloxacin (a derivative of fluoroquinolones) in poultry has resulted in emergence of resistance to ciprofloxacin. Use of modern cephalosporins has contributed to the spread of methicillin-resistant *S. aureus* in pig industries in several European countries. The acquisition of resistance in methicillin-resistant *S. aureus* of animal origin has been shown to increase prevalence of infections and therapeutic failures in humans [25].

Antimicrobial resistance in food producing animals is a global problem, particularly in developing countries including Nigeria. It is the most impenetrablequestion of public health importance. Monitoring the emergence and spread of resistant pathogens in animal reservoirs is important, particularly for those with zoonotic potential throughout the populations. The use of antimicrobial agents on dairy farms as well as in other food animal production systems is a major concern in the emergence of resistant zoonotic bacterial pathogens. The spread of antibiotics resistance among methicillin-resistant *Staphylococcus aureus* (MRSA) and coagulase-negative staphylococcal strains in dairy cattle may present a hazard for human health via food chain or through direct transmission of resistant pathogens between humans and animals.

It should be noted that all isolates that were resistant to the action of methicillin when the disc diffusion method was employed also harboured the *mec*A gene when confirmed using the PCR. Methicillin resistance is of particular relevance because it is conferred by the presence of the *mec*A gene, which encodes for production of an altered penicillin binding protein (PBP2a or PBP2′) that has a low affinity for all β-lactam antimicrobials. The *mec*A gene resides on a staphylococcal chromosomal cassette (SCC*mec*) [19]. Panton-Valentine Leucocidinis (PVL) is a pore forming toxin which damages neutrophils. It has been epidemiologically linked to primary skin and soft tissue infections and to deep-seated infections such as necrotizing pneumonia and severe recurrent osteomyelitis occurring in young immuno-competent human and animal species [26]. The presence of the *mec*A and PVL genes in the staphylococcal strains investigated in this study is very significant to public health such that they could transfer these attributes of pathogenicity to other staphylococcal strains via horizontal gene transfer. This could result in the dissemination of environmental strains with inherent capacity to resist the action of multiple antibiotics, ability to cleave the phosphodiester bonds of nucleic acids, and also render severe damage to the most important white blood cells which fights against infectious diseases by foreign invaders directly. Methicillin-resistant *Staphylococcus* species have not previously been characterized using PCR by detecting *mec*A and PVL genes signatures in food-producing animals in a rural community (Okada environs) Edo State, Nigeria. Although the extent of this problem in the rural community is currently unknown, this finding may have important implications for the nasal carriage and raw milk status of methicillin-resistant staphylococcal strains. Occurrence of antibiotic resistance in various staphylococcal strains-which in the last two decades have emerged as significant pathogens both in human and veterinary medicine-may cause problems due to the risk of horizontal transfer of antibiotic resistance determinants to commensals or pathogenic bacteria. Therefore, the presence of virulence factors and antimicrobial resistance detected in these strains should not be underestimated. However, it should be noted that the dairy cows investigated in this study are free-range and, as such, they are led by Fulani herdsmen for grazing purposes. Though these cows in question are treated with antibiotics by the local farmers rather than veterinarians whenever symptoms of illness arise, the extent and types of antibiotics used in treating them cannot be ascertained. It is also interesting to know that combined therapy ofapplying different/multiple antibiotics subcutaneously, or orally by incorporating them to their feed and water was key to the treatment of these cows. Prior to the distribution/consumption of raw milk from dairy cows, it is therefore advised that flash pasteurization be carried to reduce/remove the multiple drug resistance and virulent staphylococcal strains that might be of significance to public health.

## 5. Conclusions

This is the first report of *mecA* and PVL genes signatures that have been detected in food-producing animals in rural community Edo State, Nigeria. Furthermore, our study also demonstrated higher individual and multiple antibiotic resistance of staphylococcal strains in the rural community. These evidences highlights that the virulence potential, antibiotic resistance and the epidemiologic importance of staphylococcal strains must be further investigated. The drug resistance spectrum study provides a scientific basis for guiding the rational clinical use of antibiotics and for preventing and controlling the spread of drug-resistant bacteria.

## Figures and Tables

**Table 1:** Species diversity of staphylococcal isolates recovered from nasal and milk samples.

Staphylococcal Isolates	Nasal Isolates *n* = 50	Raw Milk Isolates *n* = 50	Total *n* = 100	*p*-Value
*S. aureus*	19 (38)	11 (22)	30 (30)	0.188
*S. chromogenes*	0 (0)	8 (16)	8 (8)	0.015
*S. epidermidis*	13 (26)	4 (8)	17 (17)	0.188
*S. haemolyticus*	0 (0)	15 (30)	15 (15)	0.225
*S. simulans*	0 (0)	7 (14)	7 (7)	0.250
*S. saprophyticus*	8 (16)	5 (10)	13 (13)	0.580
*S. pseudintermedius*	6 (12)	0 (0)	6 (6)	0.184
*S. xylosus*	4 (8)	0 (0)	4 (4)	0.184

Value in parenthesis represent (%).

**Table 2:** Antimicrobial susceptibility profile of staphylococcal isolates.

Antibiotics	Resistant Profile (%)								*p*-Value
	*S. aureus n* = 30	*S. chromogenes n* = 8	*S. epidermidis n* = 17	*S. haemolyticus n* = 15	*S. simulans n* = 7	*S. saprophyticus n* = 13	*S. pseudintermedius n* = 6	*S. xylosus n* = 4	
MET	100	100	100	100	100	100	100	100	1.000
PEN	100	100	100	100	100	100	100	100	1.000
CLX	100	100	100	100	100	92.30	100	75	0.043
AMX	100	100	100	100	100	100	100	100	1.000
ERY	90	87.50	88.20	100	100	100	83.33	100	0.645
GEN	0	0	11.76	13.33	0	0	0	0	0.015
KAN	90	100	94.12	100	100	92.31	100	100	0.794
CLN	100	100	100	100	100	100	100	100	1.000
CHL	100	100	100	100	100	100	100	100	1.000
SXT	100	100	100	100	100	100	100	100	1.000
VAN	13.30	25	5.88	26.67	57.1	7.69	0	0	0.000

**Legend**: MET: Methicillin; PEN: Penicillin; AMX: Amoxicillin; CLX: Cloxacillin; ERY: Erythromycin; GEN: Gentamycin; CLN: Clindamycin; CHL: Chloramphenicol; SXT: Trimethoprim-Sulfamethaxazole; VAN: Vancomycin; KAN: Kanamycin.

## Supplementary Materials

The following are available online at www.mdpi.com/2306-7381/3/3/14/s1, Table S1: Antimicrobial agents, disc content and zone diameter interpretative standards for *Staphylococcus* species, Table S2: Staphylococci species isolated from nasal samples, Table S3: Staphylococci species isolated from raw milk samples.
