# Supplementary material for: Detection of Methicillin-Resistant Staphylococci Isolated from Food Producing Animals: A Public Health Implication
Source: Vet Sci. 2016 Jul 4;3(3):14. doi: 10.3390/vetsci3030014 (PMC5606580; doi:10.3390/vetsci3030014)
Supplement: Supplementary file 1 [file vetsci-03-00014-s001.pdf]

Supplementary Materials: Detection of Methicillin-Resistant Staphylococci Isolated from Food Producing Animals: A Public Health Implication

Etinosa O. Igbinosa, Abeni Beshiru, Lucy U. Akporehe and Abraham G. Ogofure

Table S1. Antimicrobial agents, disc content and zone diameter interpretative standards for *Staphylococcus* species.

Antibiotics Group	Antimicrobial Agent	Disc Content	Zone Diameter (mm)		
			Resistant	Intermediate	Sensitive
Penicillins	Methicillin	5 μg	≤9	10–13	≥14
	Cloxacillin	5 μg	≤10	11–12	≥13
	Penicillin	10 μg	≤28	-	≥29
	Amoxicillin	10 μg	≤19	-	≥20
Macrolides	Erythromycin	15 μg	≤13	14–22	≥23
Aminoglycosides	Gentamycin	10 μg	≤12	13–14	≥15
	Kanamycin	30 μg	≤13	14–17	≥18
Lincosamides	Clindamycin	2 μg	≤14	15–20	≥21
Phenicols	Chloramphenicol	30 μg	≤12	13–17	≥18
Folates	Trimethoprim-Sulfamethoxazole	1.25 μg	≤10	11–15	≥16
Glycopeptides	Vancomycin	30 μg	-	-	≥15

Source: CLSI [1].

Table S2. Staphylococci species isolated from nasal samples.

Isolates Code	Identification	Max Score	Total Score	Query Cover %	E-Value	Identity %	Accession
N001	*Staphylococcus saprophyticus*	97.6	97.6	100%	1E − 17	100%	KT720173.1
N002	*Staphylococcus intermedius*	97.6	97.6	100%	1E − 17	100%	FN646071.1
N003	*Staphylococcus saprophyticus*	97.6	97.6	100%	1E − 17	100%	KR709233.1
N004	*Staphylococcus intermedius*	97.6	97.6	100%	1E − 17	100%	FN646071.1
N005	*Staphylococcus saprophyticus*	97.6	97.6	100%	1E − 17	100%	KT719854.1
N006	*Staphylococcus saprophyticus*	97.6	97.6	100%	1E − 17	100%	KT720164.1
N007	*Staphylococcus saprophyticus*	97.6	97.6	100%	1E − 17	100%	KT720168.1
N008	*Staphylococcus aureus*	97.6	97.6	100%	1E − 17	100%	FN646074.1
N009	*Staphylococcus aureus*	97.6	97.6	100%	1E − 17	100%	FN646074.1
N010	*Staphylococcus saprophyticus*	97.6	97.6	100%	1E − 17	100%	KT720171.1
N011	*Staphylococcus epidermidis*	89.7	89.7	100%	3E − 15	98%	CP009046.1
N012	*Staphylococcus saprophyticus*	97.6	97.6	100%	1E − 17	100%	KT720172.1
N013	*Staphylococcus intermedius*	97.6	97.6	100%	1E − 17	100%	FN646071.1
N014	*Staphylococcus epidermidis*	89.7	89.7	100%	3E − 15	98%	CP009046.1
N015	*Staphylococcus epidermidis*	89.7	89.7	100%	3E − 15	98%	CP009046.1
N016	*Staphylococcus aureus*	97.6	97.6	100%	1E − 17	100%	FN646074.1
N017	*Staphylococcus aureus*	97.6	97.6	100%	1E − 17	100%	FN646074.1
N018	*Staphylococcus aureus*	97.6	97.6	100%	1E − 17	100%	FN646074.1
N019	*Staphylococcus aureus*	97.6	97.6	100%	1E − 17	100%	FN646074.1
N020	*Staphylococcus aureus*	97.6	97.6	100%	1E − 17	100%	FN646074.1
N021	*Staphylococcus aureus*	97.6	97.6	100%	1E − 17	100%	FN646074.1
N022	*Staphylococcus aureus*	75.8	75.8	93%	4E − 11	96%	KR028437.1
N023	*Staphylococcus aureus*	75.8	75.8	93%	4E − 11	96%	KR028437.1
N024	*Staphylococcus aureus*	75.8	75.8	93%	4E − 11	96%	KR028437.1
N025	*Staphylococcus aureus*	97.6	97.6	100%	1E − 17	100%	FN646074.1
N026	*Staphylococcus epidermidis*	89.7	89.7	100%	3E − 15	98%	CP009046.1
N027	*Staphylococcus aureus*	97.6	97.6	100%	1E − 17	100%	FN646074.1
N028	*Staphylococcus aureus*	97.6	97.6	100%	1E − 17	100%	FN646074.1
N029	*Staphylococcus epidermidis*	89.7	89.7	100%	3E − 15	98%	FJ577634.1

Table S2. *Cont.*

Isolates Code	Identification	Max Score	Total Score	Query Cover %	E-Value	Identity %	Accession
N030	*Staphylococcus saprophyticus*	97.6	97.6	100%	1E − 17	100%	KT720173.1
N031	*Staphylococcus epidermidis*	89.7	89.7	100%	3E − 15	98%	EU659874.1
N032	*Staphylococcus epidermidis*	89.7	89.7	100%	3E − 15	98%	EU659874.1
N033	*Staphylococcus aureus*	75.8	75.8	93%	4E − 11	96%	KR028437.1
N034	*Staphylococcus intermedius*	97.6	97.6	100%	1E − 17	100%	FN646071.1
N035	*Staphylococcus epidermidis*	89.7	89.7	100%	3E − 15	98%	FJ577634.1
N036	*Staphylococcus intermedius*	97.6	97.6	100%	1E − 17	100%	FN646071.1
N037	*Staphylococcus epidermidis*	89.7	89.7	100%	3E − 15	98%	FJ577634.1
N038	*Staphylococcus aureus*	97.6	97.6	100%	1E − 17	100%	FN646074.1
N039	*Staphylococcus epidermidis*	89.7	89.7	100%	3E − 15	98%	FJ577634.1
N040	*Staphylococcus epidermidis*	89.7	89.7	100%	3E − 15	98%	FJ577634.1
N041	*Staphylococcus aureus*	97.6	97.6	100%	1E − 17	100%	FN646074.1
N042	*Staphylococcus aureus*	97.6	97.6	100%	1E − 17	100%	FN646074.1
N043	*Staphylococcus epidermidis*	89.7	89.7	100%	3E − 15	98%	FJ577634.1
N044	*Staphylococcus epidermidis*	89.7	89.7	100%	3E − 15	98%	FJ577634.1
N045	*Staphylococcus aureus*	97.6	97.6	100%	1E − 17	100%	FN646074.1
N046	*Staphylococcus intermedius*	97.6	97.6	100%	1E − 17	100%	FN646071.1
N047	*Staphylococcus xylosus*	97.6	97.6	100%	1E − 17	100%	KT351728.1
N048	*Staphylococcus xylosus*	97.6	97.6	100%	1E − 17	100%	KT021502.1
N049	*Staphylococcus xylosus*	97.6	97.6	100%	1E − 17	100%	KR856344.1
N050	*Staphylococcus xylosus*	97.6	97.6	100%	1E − 17	100%	KP668813.1

Table S3. Staphylococci species isolated from raw milk samples.

Isolates Code	Identification	Max Score	Total Score	Query Cover %	E-Value	Identity %	Accession
M001	*Staphylococcus chromogenes*	93.7	93.7	95%	2E − 16	100%	KC481609.1
M002	*Staphylococcus chromogenes*	93.7	93.7	95%	2E − 16	100%	KC481609.1
M003	*Staphylococcus chromogenes*	97.6	97.6	100%	1E − 17	100%	EU622581.1
M004	*Staphylococcus chromogenes*	97.6	97.6	100%	1E − 17	100%	EU622581.1
M005	*Staphylococcus epidermidis*	89.7	89.7	100%	3E − 15	98%	FJ577582.1
M006	*Staphylococcus epidermidis*	89.7	89.7	100%	3E − 15	98%	HG813242.1
M007	*Staphylococcus epidermidis*	89.7	89.7	100%	3E − 15	98%	HG813242.1
M008	*Staphylococcus aureus*	97.6	97.6	100%	1E − 17	100%	FN646074.1
M010	*Staphylococcus haemolyticus*	97.6	97.6	100%	1E − 17	100%	KT696497.1
M011	*Staphylococcus haemolyticus*	97.6	97.6	100%	1E − 17	100%	KP324951.1
M012	*Staphylococcus aureus*	97.6	97.6	100%	1E − 17	100%	FN646074.1
M013	*Staphylococcus aureus*	97.6	97.6	100%	1E − 17	100%	FN646074.1
M014	*Staphylococcus haemolyticus*	97.6	97.6	100%	1E − 17	100%	KP324951.1
M015	*Staphylococcus haemolyticus*	97.6	97.6	100%	1E − 17	100%	KT696497.1
M016	*Staphylococcus haemolyticus*	97.6	97.6	100%	1E − 17	100%	KT696497.1
M017	*Staphylococcus haemolyticus*	97.6	97.6	100%	1E − 17	100%	KT696497.1
M018	*Staphylococcus aureus*	97.6	97.6	100%	1E − 17	100%	FN646074.1
M019	*Staphylococcus haemolyticus*	97.6	97.6	100%	1E − 17	100%	KP324951.1
M020	*Staphylococcus haemolyticus*	97.6	97.6	100%	1E − 17	100%	KP324951.1
M021	*Staphylococcus aureus*	97.6	97.6	100%	1E − 17	100%	FN646074.1
M022	*Staphylococcus aureus*	97.6	97.6	100%	1E − 17	100%	FN646074.1
M023	*Staphylococcus haemolyticus*	97.6	97.6	100%	1E − 17	100%	KP324951.1
M024	*Staphylococcus haemolyticus*	97.6	97.6	100%	1E − 17	100%	KP324951.1
M025	*Staphylococcus saprophyticus*	97.6	97.6	100%	1E − 17	100%	KT720164.1
M026	*Staphylococcus epidermidis*	89.7	89.7	100%	3E − 15	98%	FJ577582.1
M027	*Staphylococcus saprophyticus*	97.6	97.6	100%	1E − 17	100%	KT720168.1
M028	*Staphylococcus chromogenes*	93.7	93.7	95%	2E − 16	100%	KC481609.1
M029	*Staphylococcus chromogenes*	93.7	93.7	95%	2E − 16	100%	KC481609.1
M030	*Staphylococcus simulans*	93.7	93.7	100%	2E − 16	100%	FN646077.1

Table S3. *Cont.*

Isolates Code	Identification	Max Score	Total Score	Query Cover %	E-Value	Identity %	Accession
M031	*Staphylococcus aureus*	97.6	97.6	100%	1E − 17	100%	FN646074.1
M032	*Staphylococcus simulans*	93.7	93.7	100%	2E − 16	100%	FN646077.1
M033	*Staphylococcus simulans*	93.7	93.7	100%	2E − 16	100%	FN646077.1
M034	*Staphylococcus simulans*	93.7	93.7	100%	2E − 16	100%	FN646077.1
M035	*Staphylococcus aureus*	97.6	97.6	100%	1E − 17	100%	FN646074.1
M036	*Staphylococcus haemolyticus*	97.6	97.6	100%	1E − 17	100%	KP324951.1
M037	*Staphylococcus haemolyticus*	97.6	97.6	100%	1E − 17	100%	KP324951.1
M038	*Staphylococcus simulans*	93.7	93.7	100%	2E − 16	100%	FN646077.1
M039	*Staphylococcus saprophyticus*	97.6	97.6	100%	1E − 17	100%	KT720173.1
M040	*Staphylococcus saprophyticus*	97.6	97.6	100%	1E − 17	100%	KT720172.1
M041	*Staphylococcus chromogenes*	97.6	97.6	100%	1E − 17	100%	EU622581.1
M042	*Staphylococcus aureus*	97.6	97.6	100%	1E − 17	100%	FN646074.1
M043	*Staphylococcus haemolyticus*	97.6	97.6	100%	1E − 17	100%	KP324951.1
M044	*Staphylococcus haemolyticus*	97.6	97.6	100%	1E − 17	100%	KP324951.1
M045	*Staphylococcus simulans*	93.7	93.7	100%	2E − 16	100%	FN646077.1
M046	*Staphylococcus aureus*	97.6	97.6	100%	1E − 17	100%	FN646074.1
M047	*Staphylococcus simulans*	93.7	93.7	100%	2E − 16	100%	FN646077.1
M048	*Staphylococcus chromogenes*	97.6	97.6	100%	1E − 17	100%	EU622581.1
M049	*Staphylococcus aureus*	97.6	97.6	100%	1E − 17	100%	FN646074.1
M050	*Staphylococcus saprophyticus*	97.6	97.6	100%	1E − 17	100%	KT720171.1

References

1. *Performance Standards for Antimicrobial Susceptibility Testing; Sixteen International Supplements*; Clinical and Laboratory Standards Institute: Wayne, PA, USA, 2006; p. 118-123.

© 2016 by the authors; licensee MDPI, Basel, Switzerland. This article is an open access article distributed under the terms and conditions of the Creative Commons by Attribution (CC-BY) license (http://creativecommons.org/licenses/by/4.0/).
